# Supplementary material for: A theoretical and experimental framework enables low-coverage sequencing for accurate quantification of genome-wide cytosine modification levels
Source: NAR Genom Bioinform. 2026 Jul 28;8(3):lqag082. doi: 10.1093/nargab/lqag082 (PMC13408044; doi:10.1093/nargab/lqag082)
Supplement: lqag082_Supplemental_Files [file lqag082_supplemental_files.zip › SI-Clean.pdf]

SUPPLEMENTARY INFORMATION

A theoretical and experimental framework enables low-coverage sequencing for accurate quantification of genome-wide cytosine modification levels

Christian E. Loo[1], Johanna M. Fowler[2], Heqiao Zhu[3,4], Christopher Krapp[5], Ruiyao Zhu[2], Marisa S. Bartolomei[5], Wanding Zhou[3,4‡], Rahul M. Kohli[2‡]

‡ Correspondence to: Wanding Zhou (wanding.zhou@pennmedicine.upenn.edu), Rahul M. Kohli (rkohli@pennmedicine.upenn.edu)

[1]Graduate Group in Biochemistry, Biophysics, and Chemical Biology, University of Pennsylvania, Philadelphia, PA, USA
[2]Department of Medicine, University of Pennsylvania, Philadelphia, PA, USA
[3]Center for Computational and Genomic Medicine, Children's Hospital of Philadelphia, PA, USA
[4]Department of Pathology and Laboratory Medicine, University of Pennsylvania, Philadelphia, PA, USA
[5]Department of Cell and Developmental Biology, University of Pennsylvania, Philadelphia, PA, USA

Supplementary Figures

Figure S1. **Computational downsampling applied to published ACE-Seq datasets**
Figure S2. **Computational downsampling applied to published EM-Seq datasets**
Figure S3. **Distribution of genomic element prevalence relative to mappable reads**
Figure S4. **Computational downsampling applied to genomic element analysis for whole-genome datasets**
Figure S5. **Total Analytical Error (TAE) for 5mCpH and 5hmCpG measurements across murine development**
Figure S6. **Ternary plots for change in DNA modifications by genomic element over development**
Figure S7. **Total Analytical Error (TAE) for unmodified CpG, 5mCpG, and 5hmCpG measurements at transcribed elements across murine development**
Figure S8. **Comparison of methods for quantifying cytosine modifications**

Supplementary Tables

Table S1. **Computational downsampling and derived Total Analytical Error (TAE)**
Table S2. **Total Analytical Error (TAE) as a function of genome coverage and % cytosine modification level**

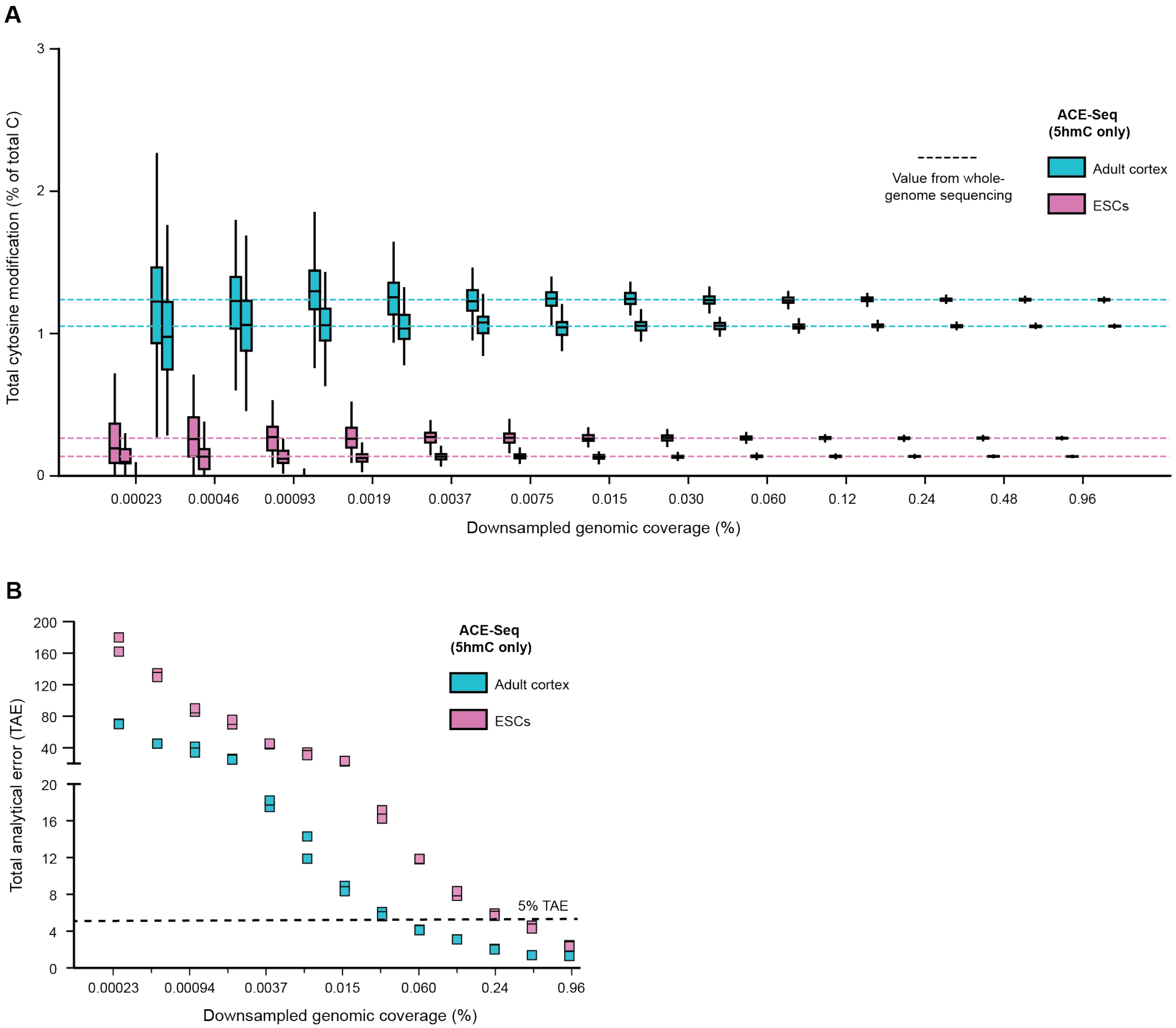

Figure S1. Computational downsampling applied to published ACE-Seq datasets. A) Whole-genome sequencing data sets sequenced with ACE-Seq in various murine cell types were downsampled to sparse genomic coverage. Box plots represent the cytosine modification levels determined in each of 100 replicates at each sparse coverage level. Dashed lines mark the cytosine modification levels from the complete, deeply sequenced whole-genome dataset. **B)** Shown is the calculated total analytical error (TAE) as a function of the downsampled coverage from the whole-genome datasets.

A

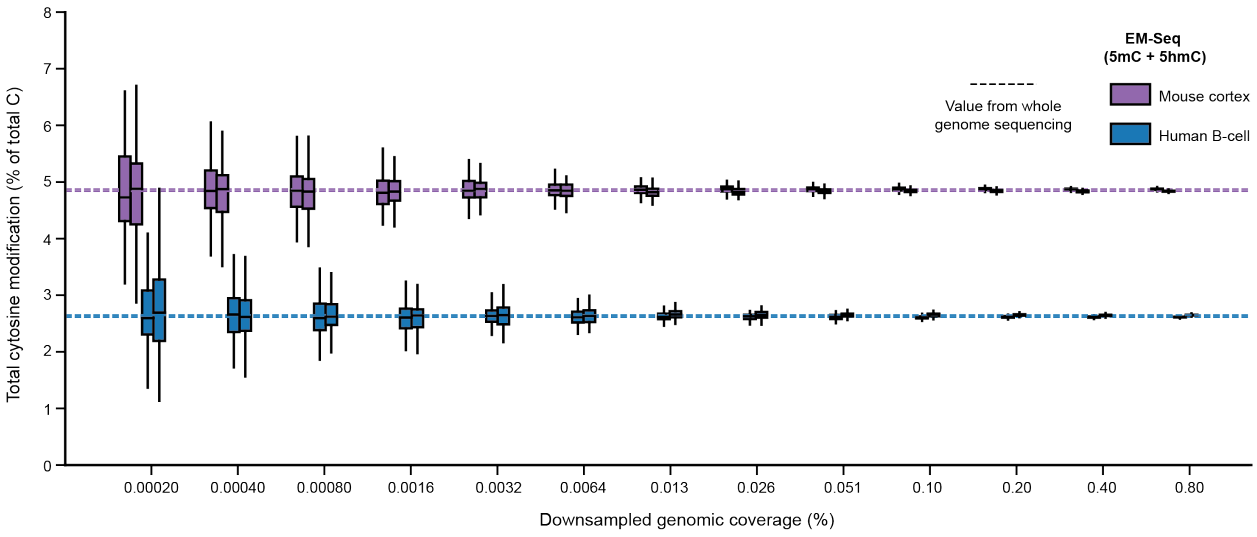

B

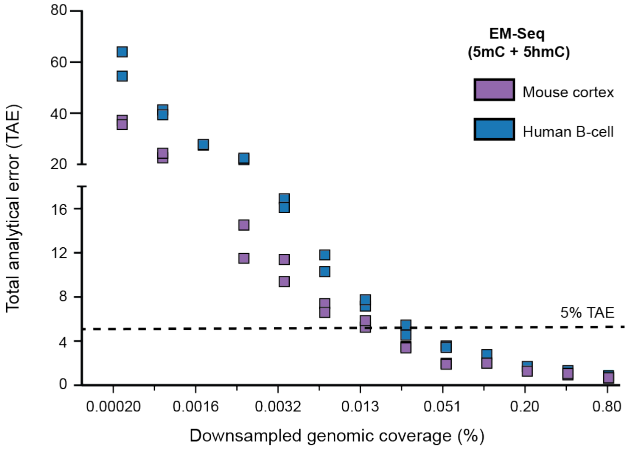

Figure S2. Computational downsampling applied to published EM-Seq datasets. A) Whole-genome sequencing data sets sequenced with EM-Seq in various murine cell types were downsampled to sparse genomic coverage. Box plots represent the cytosine modification levels determined in each of 100 replicates at each sparse coverage level. Dashed lines mark the cytosine modification levels from the full, deeply sequenced whole-genome dataset. **B)** Shown is the calculated total analytical error (TAE) as a function of the downsampled coverage from the whole-genome datasets.

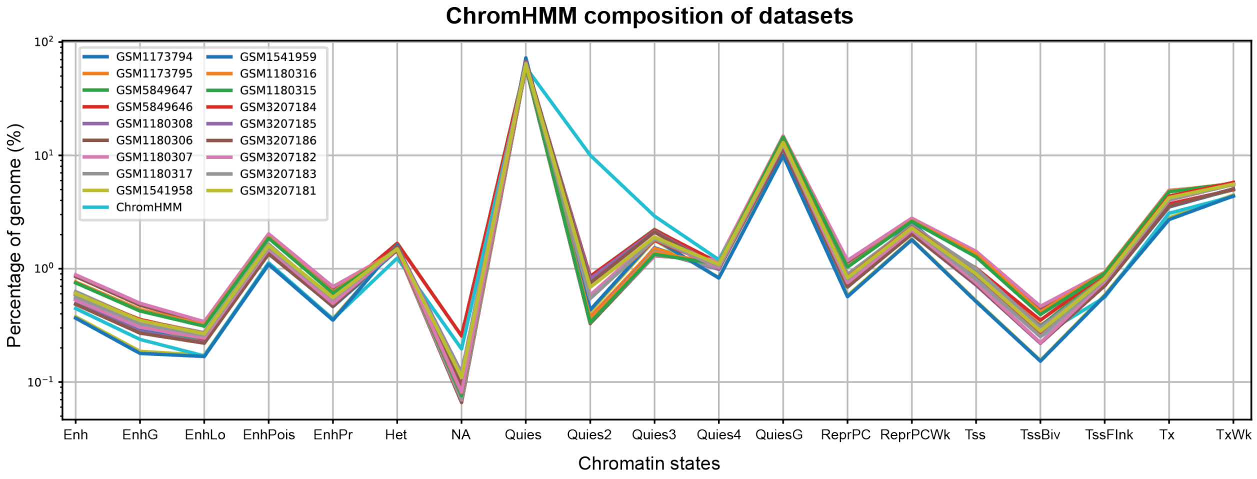

Figure S3. Distribution of genomic element prevalence relative to mappable reads. Using various genomic elements categorized by ChromHMM, plotted in light blue is the percentage of the genome (log-scale) attributable to a given genomic element. For the other deeply sequenced, whole-genome datasets, plotted are the percentage of reads that uniquely map to a given genomic element. While fewer reads map to quiescent regions, for other genomic elements the percentage of reads mapped from deeply sequenced data sets generally tracks with their abundance in the genome.

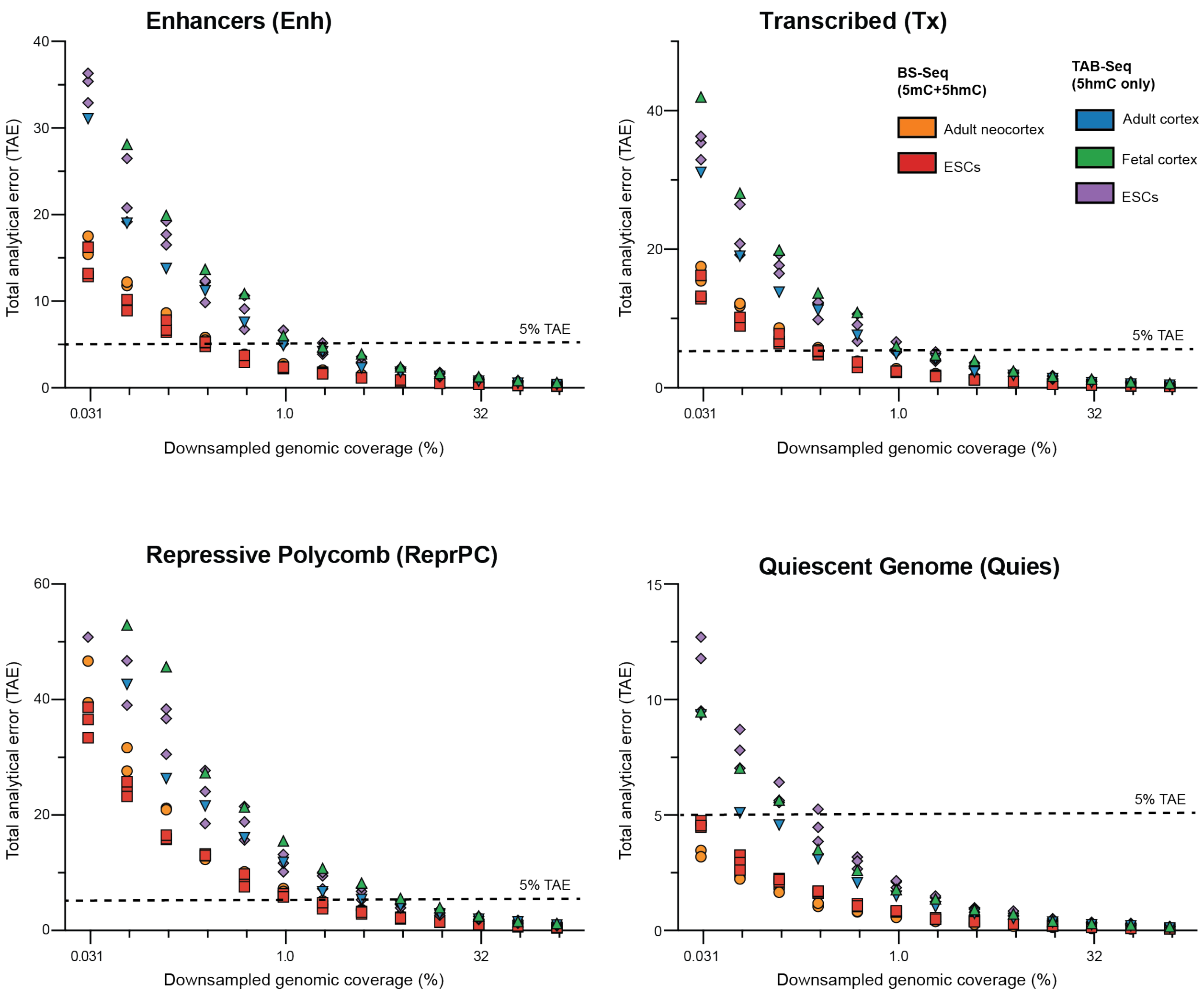

Figure S4. Computational downsampling applied to genomic element analysis for whole-genome datasets. Whole-genome sequencing datasets generated from various methods were downsampled to sparse genomic coverage. The resulting reads were mapped and categorized by ChromHMM element, correlating with different common genomic elements. Shown is the calculated total analytical error (TAE) as a function of the downsampled coverage for various genomic elements.

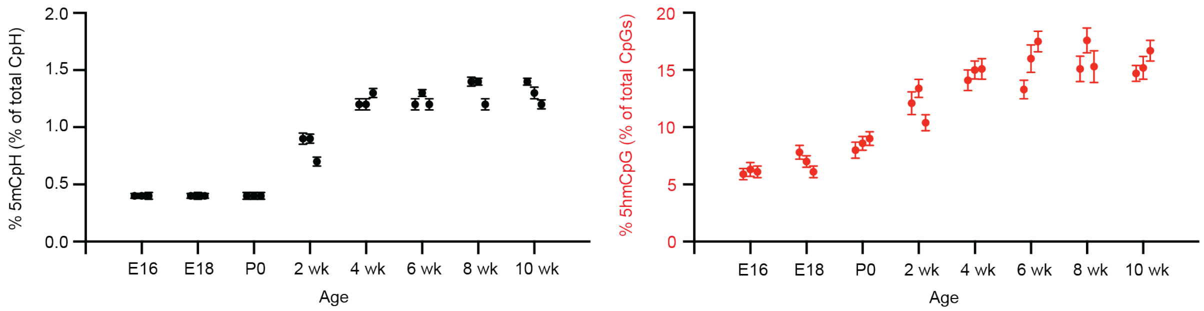

Figure S5. Total Analytical Error (TAE) for 5mCpH and 5hmCpG measurements across murine development. Shown are plots of 5mCpH (left) and 5hmCpG (right) levels (%) in the developing mouse brain detected by Sparse BS/bACE-Seq. At each timepoint, three data points are shown, each representing an independent biological replicate. TAE bars derived from the TAE calculator are shown for each datapoint.

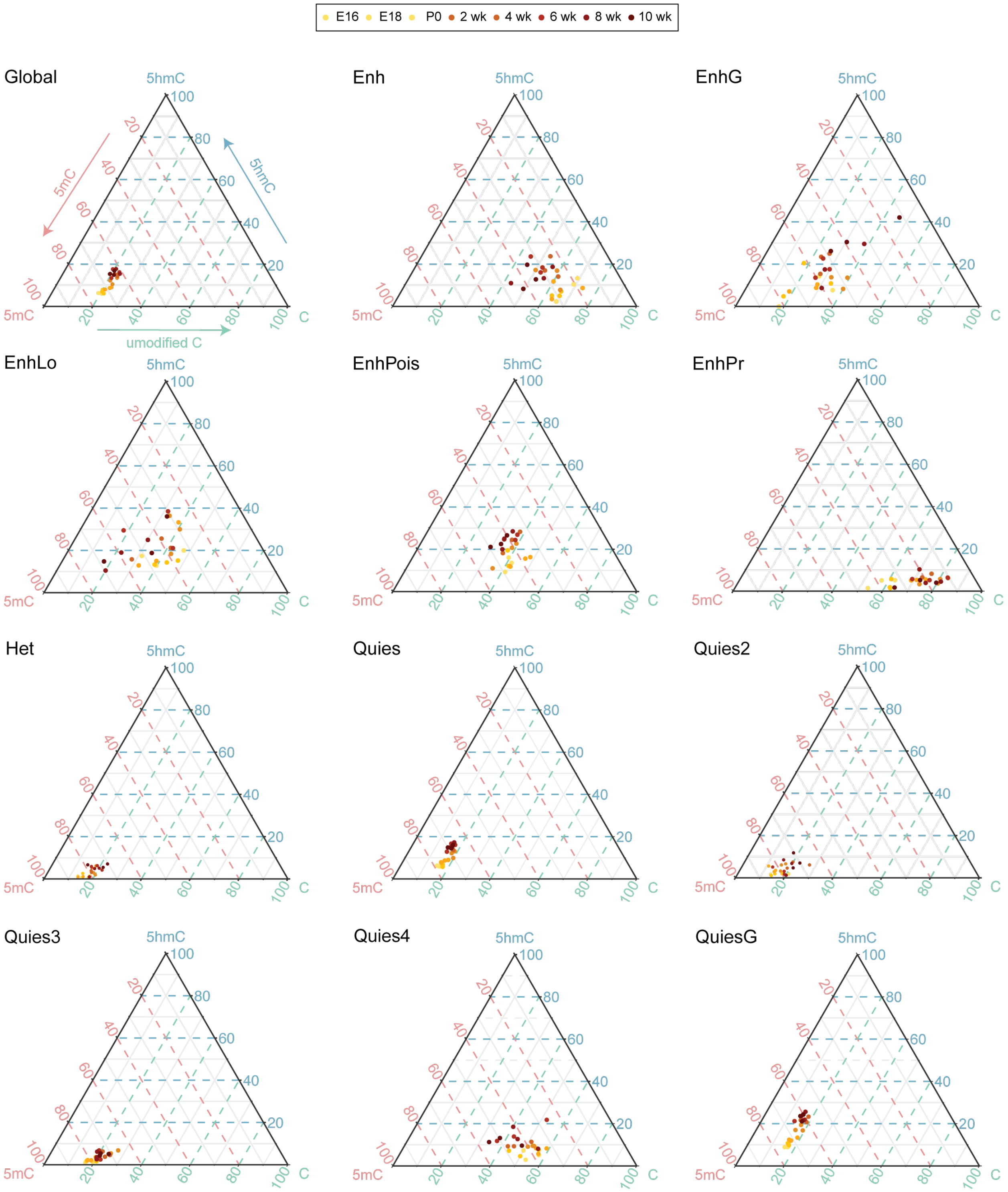
E16 E18 P0 2 wk 4 wk 6 wk 8 wk 10 wk
Global
Enh
EnhG
EnhLo
EnhPois
EnhPr
Het
Quies
Quies2
Quies3
Quies4
QuiesG
5hmC
5mC
C
umodified C

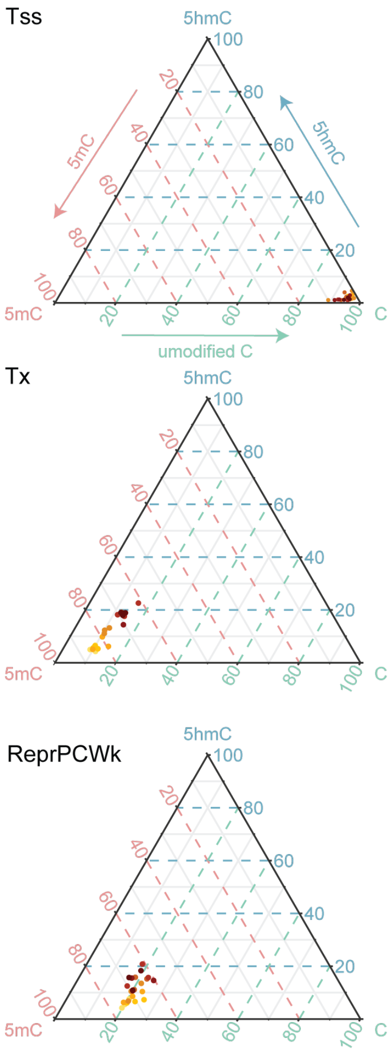

Figure S6. Ternary plots for change in DNA modifications by genomic element over development. Shown are ternary plots representing the levels of C, 5mC, and 5hmC measured for the whole genome or specific genomic elements. Each data point represents the measurement from a single biological replicate, with different time points represented by different colors.

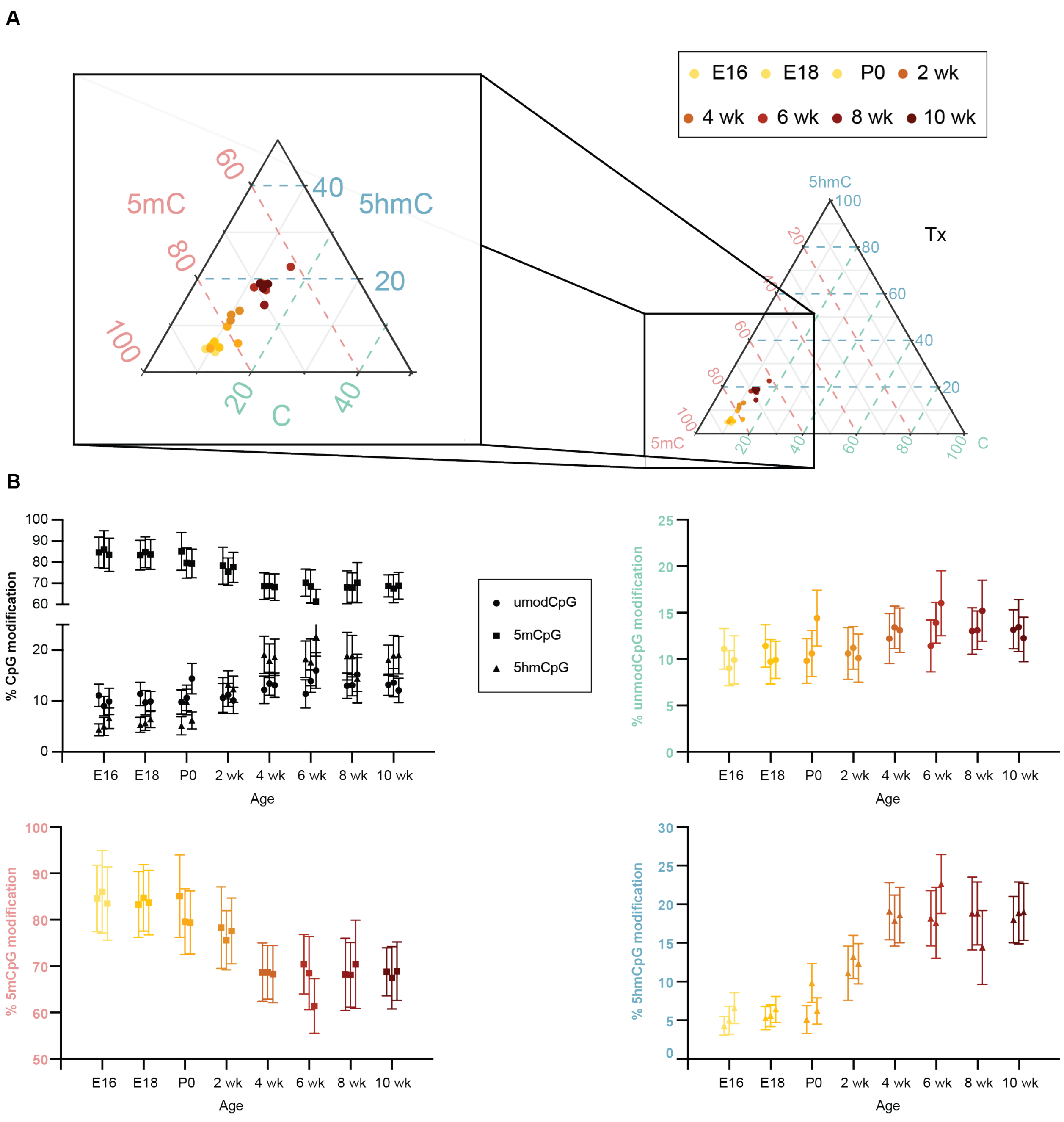

Figure S7. Total Analytical Error (TAE) for unmodified CpG, 5mCpG, and 5hmCpG measurements at transcribed elements across murine development. A) A ternary plot of the Tx chromatin state across the murine brain development, detected by Sparse BS/bACE-Seq, reveals that unmodified CpG levels remain stable while 5mCpG levels decrease and 5hmC levels increase over time. A zoom in of the relevant region of the ternary plot is shown. **B)** These dynamics are illustrated in scatter plots showing unmodified CpG, 5mCpG, and 5hmCpG plotted together (top left), unmodified CpG alone (top right), 5mCpG alone (bottom left), and 5hmCpG (bottom right). At each timepoint, three data points are shown, each representing an independent biological replicate. TAE bars derived from the TAE calculator are shown for each dataset, allowing for determination of trends that account for measurement error.

	LC-MS/MS	Array-Based Methods (e.g., EPIC BeadChip)	Deep Whole-Genome Sequencing	Sparse Sequencing
**Core Principle**	Genomic DNA is enzymatically degraded to individual nucleosides, separated by liquid chromatography, and detected by tandem mass spectrometry quantified against standard curves for individual nucleosides	**Shared Principle:** Genomic DNA is converted, either chemically or enzymatically, to discriminate modified from unmodified cytosines		
		**Differentiated Principle:** Converted library hybridizes to arrays querying preselected CpG probe sites, fluorescence intensity reports on methylation state of the preselected sites	**Differentiated Principle:** NGS resolves modification status at every cytosine position, genome-wide, at high read depth (e.g., 10-30x)	**Differentiated Principle:** Converted library is sequenced to shallow depth, modification levels are estimated either genome-wide or at elements with a tunable TAE
**Key Features**	• Quantifies absolute molar amounts of modified cytosine states<br>• Highly specific MS/MS transitions<br>• Independent of sequence contexts and genomic features	• Covers hundreds of thousands of CpG sites<br>• Provides position-level, but not global, methylation estimates<br>• Primarily designed for 5mC (5hmC coverage not well established)<br>• Poor resolution at very low (<5%) or high (>95%) modification levels	• Single-base resolution across the genome<br>• At high depth can resolve low level modifications	• Accurate genome-wide quantification from just tens of thousands of mapped reads
			• Retains full sequence context (e.g., CpG, CpH)<br>• Can separately quantify 5mC and 5hmC with paired methods (e.g., BS, bACE)<br>• Provides complete positional information (mapping to genomic elements)	
**DNA Input**	>500 ng; standard, efforts to reduce exist, but add complexity	250 ng - 500 ng; standard, though lower-input protocols exist	10 ng (enzymatic) - 1 µg (BS), though sub ng input protocols exist	10 ng (enzymatic) - 1 µg (BS), though sub ng input protocols exist
**Cost**	Specialized LC-MS/MS instrument required; expensive to establish and operate, access is not universal	DNA conversion and sample preparation costs are the same between these methods		
		**Sequencing costs:** Moderate (~**$300/sample**, requires array scanner)	**Sequencing costs:** High (up to **$1000/sample** for 30x WGBS, computation-intensive)	**Sequencing costs:** Low (**~$3/sample** with a MiSeq v3 600 cycle kit, optimal multiplexing)
**Throughput**	Low-to-moderate: manually time intensive, samples analyzed one at a time	**High**: scalable across large cohorts	**Low**: not feasible for large cohorts at base resolution	**High**: scalable across large cohorts
**Limitations**	• DNA is degraded; sequence context and genomic location for modifications is lost<br>• Requires access to LC-MS/MS instrumentation and expertise; low throughput<br>• Multiple standard curves can compound error when comparing between modifications	• Pre-selected probe sites may introduce bias for global estimations<br>• No positional flexibility, cannot query non-targeted genomic elements<br>• Not ideal for profiling non-CpG methylation or 5hmC<br>• Reference panels are human-centric, limited for non-human models	• Cost-prohibitive for large cohorts<br>• Substantial computational overhead and data storage required<br>• Overkill when only global/element-level estimates are needed	• Does not provide base-resolution maps across the full genome<br>• TAE rises at exceptionally sparse modification levels<br>• Genomic element-level analyses require proportionally greater depth
**Ideal Use Cases**	• Small studies where precise molar quantification is paramount and context is irrelevant<br>• Detection of unusual or rare cytosine modifications	• Large studies studying site-specific epigenetic modification variants (e.g., epidemiological or EWAS cohorts)<br>• Studies focused on well-annotated human CpG loci	• Studies where base-resolution is absolutely required (e.g., single-cell methylome profiling)	• Studies profiling global modification levels across many samples or timepoints<br>• Studies where genomic context or element-level trends matter<br>• Pre-screening samples prior to investing in deep sequencing
**Illustrative Example**	A study needs to confirm that a TET2 KO abolishes 5hmC production in a cell line	An EWAS study of 1000 patient blood samples to identify loci associated with a disease risk	A study to identify which exact CpGs at a gene promoter switch from methylated to unmethylated during differentiation	A study is profiling global 5hmC levels as a biomarker across 200 patient cfDNA samples, where input is limited, high throughput is required, and high accuracy is needed to draw conclusions

Figure S8. Comparison of methods for quantifying cytosine modifications. Four approaches for measuring unmodified cytosine, 5mC, and 5hmC are detailed and compared across key experimental and practical dimensions. Illustrative examples of use cases for each of these methods are also noted. Abbreviations: LC-MS/MS, liquid chromatography-tandem mass spectrometry; NGS, next-generation sequencing; TAE, total analytical error; bACE-Seq, bisulfite-assisted APOBEC-coupled epigenetic sequencing; WGBS, whole-genome bisulfite sequencing; EWAS, epigenome-wide association study; cfDNA, cell-free DNA.
